# Supplementary material for: Early tuberculosis treatment remodels blood mRNA and small RNA, and a baseline host signature is associated with early culture conversion
Source: bioRxiv. 2026 Oct 27:2026.10.25.761687. Preprint. [Version 1] doi: 10.64898/2026.10.25.761687 (PMC13733870; doi:10.64898/2026.10.25.761687)
Supplement: Supplementary file 1 [file NIHPP2026.10.25.761687V1-supplement-1.pdf]

bioRxiv preprint doi: https://doi.org/10.64898/2026.10.25.761687; this version posted October 27, 2026. The copyright holder for this preprint (which was not certified by peer review) is the author/funder, who has granted bioRxiv a license to display the preprint in perpetuity. It is made available under a CC-BY 4.0 International license.

Supplementary Material

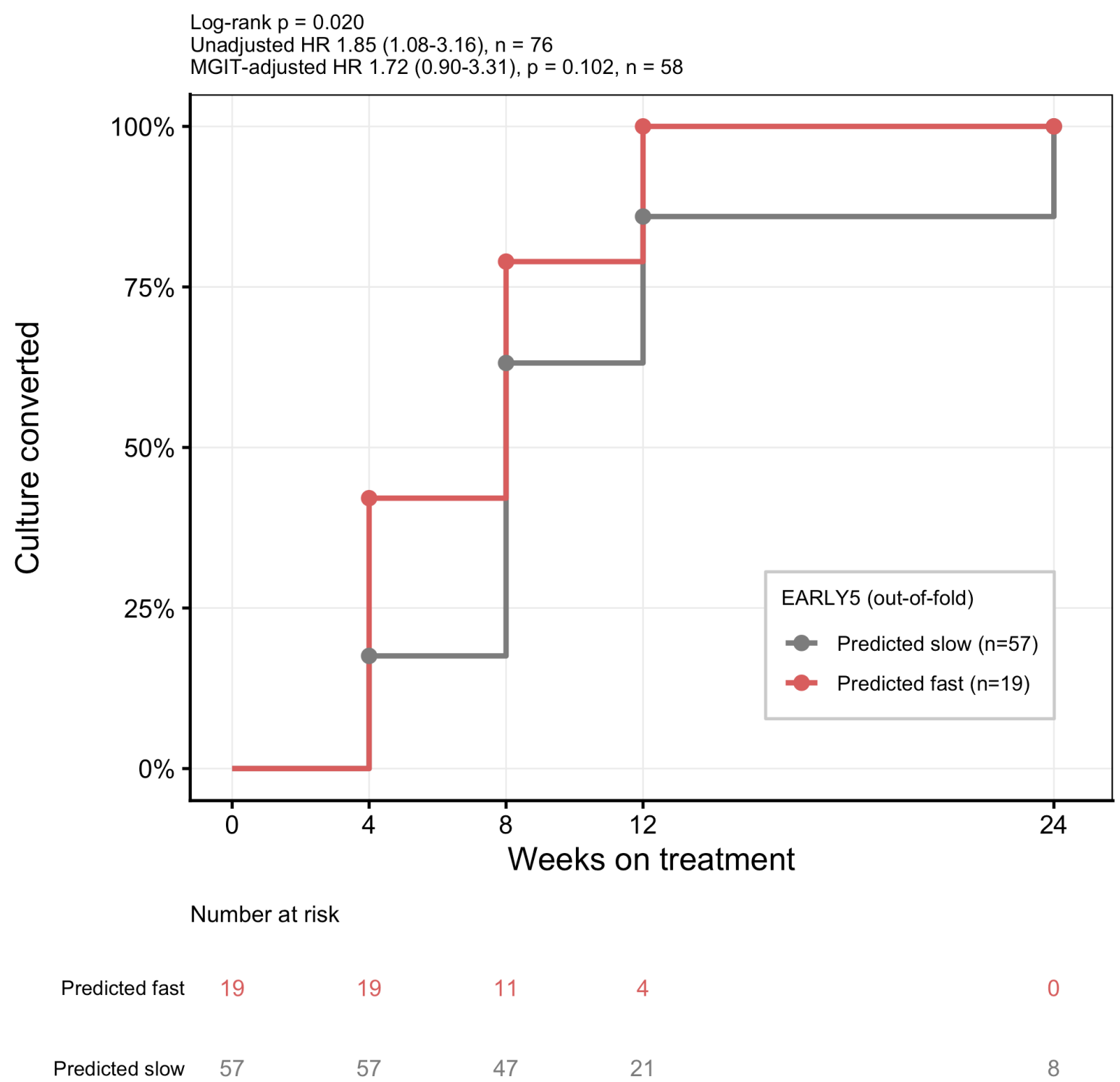

Supplementary Figure S1. Time to sputum culture conversion by EARLY5 prediction.

Cumulative proportion of participants with sputum culture conversion in GSE89403, grouped by out-of-fold EARLY5 classification (predicted fast, n = 19; predicted slow, n = 57). Participants predicted to convert fast did so earlier (log-rank p = 0.020; HR 1.85, 95% CI 1.08–3.16). After adjustment for baseline MGIT time-to-positivity, the association was attenuated (HR 1.72, 95% CI 0.90–3.31, p = 0.102; n = 58). Culture conversion was assessed at weeks 4, 8, 12 and 24. Numbers at risk are shown below the plot.

bioRxiv preprint doi: https://doi.org/10.64898/2026.10.25.761687; this version posted October 27, 2026. The copyright holder for this preprint (which was not certified by peer review) is the author/funder, who has granted bioRxiv a license to display the preprint in perpetuity. It is made available under a CC-BY 4.0 International license.

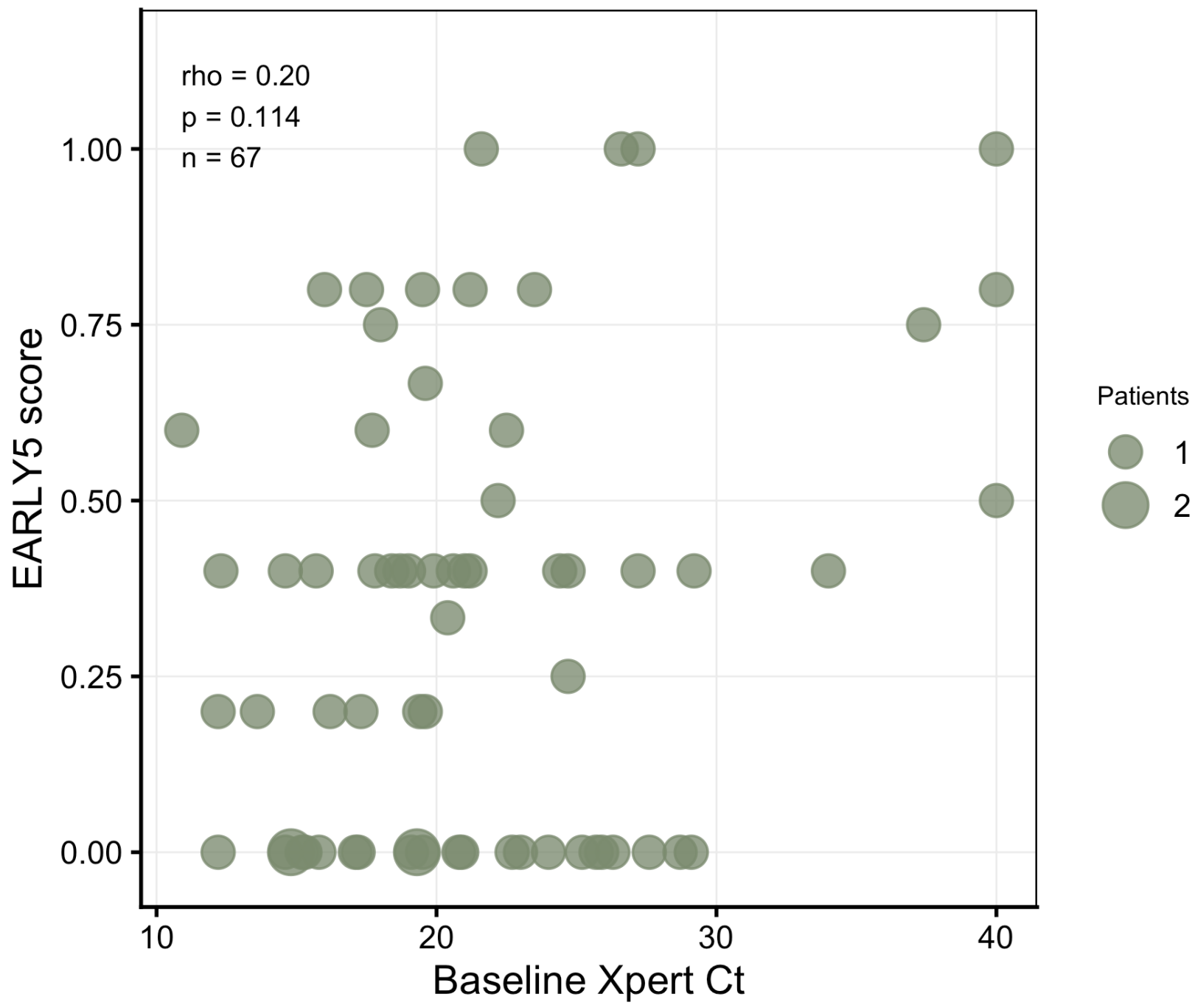

Supplementary Figure S2. EARLY5 score and baseline Xpert Ct.

EARLY5 LOOCV scores were not significantly correlated with baseline Xpert cycle threshold (n = 67; Spearman rho = 0.20, p = 0.114). Ct values of 40 indicate no detection (n = 3). Excluding these participants, the correlation was weaker (n = 64; Spearman rho = 0.10, p = 0.43). Point size reflects the number of participants at each value.

Supplementary Table S1. Meta-Analysis-derived DEGs with associated statistics.

Gene symbol, direction of change, meta-analysis log2FC, Benjamini-Hochberg adjusted p-value, raw combined p-value, combined Stouffer Z-score, number of contributing studies, directional consistency flag, study-specific log2FC and adjusted p-value for GSE89403 and the study cohort, and Human Protein Atlas immune cell type assignment. Meta-DEGs are defined by FDR < 0.05, |log2FC| ≥ 1, and directional concordance across both cohorts.

Gene symbol	Direction	Meta log_2FC	Meta FDR	Meta p	Stouffer Z	Studies	Concordant	GSE89403 log_2FC	GSE89403 FDR	Study cohort log_2FC	Study cohort FDR	HPA cell type
ATP6V0CP4	Up	1.46	1.31×10^{-15}	2.06×10^{-17}	8.49	2	Yes	1.62	1.18×10^{-15}	1.02	0.596	Other
PRSS33	Up	1.43	1.93×10^{-19}	7.36×10^{-22}	9.61	2	Yes	1.64	2.76×10^{-19}	0.84	0.568	Eosinophil
ALOX15	Up	1.33	1.79×10^{-19}	6.69×10^{-22}	9.62	2	Yes	1.64	2.15×10^{-20}	0.47	0.713	Eosinophil
LINC02073	Up	1.32	1.18×10^{-15}	1.79×10^{-17}	8.51	2	Yes	1.52	1.35×10^{-15}	0.73	0.581	lncRNA
HRK	Up	1.29	1.64×10^{-16}	1.96×10^{-18}	8.76	2	Yes	1.42	4.98×10^{-16}	0.90	0.568	B cell
MEG3	Up	1.26	2.03×10^{-13}	7.64×10^{-15}	7.77	2	Yes	1.32	1.38×10^{-12}	1.11	0.568	lncRNA
CACNG6	Up	1.22	4.91×10^{-17}	4.90×10^{-19}	8.91	2	Yes	1.30	2.22×10^{-16}	1.01	0.568	Basophil
PLVAP	Up	1.21	6.76×10^{-14}	2.15×10^{-15}	7.93	2	Yes	1.37	6.96×10^{-14}	0.76	0.608	Dendritic cell
SIGLEC8	Up	1.20	2.53×10^{-18}	1.50×10^{-20}	9.29	2	Yes	1.55	9.73×10^{-20}	0.21	0.843	Eosinophil
SFRP2	Up	1.17	9.43×10^{-13}	4.44×10^{-14}	7.55	2	Yes	1.24	2.19×10^{-12}	0.97	0.573	Other
PLAAT5	Up	1.15	5.77×10^{-18}	4.36×10^{-20}	9.18	2	Yes	1.42	4.84×10^{-19}	0.40	0.773	Eosinophil
CXCL8	Up	1.06	4.76×10^{-10}	4.86×10^{-11}	6.58	2	Yes	0.88	2.22×10^{-9}	1.58	0.568	Neutrophil
PMP22	Up	1.05	5.79×10^{-15}	1.21×10^{-16}	8.28	2	Yes	1.19	9.44×10^{-15}	0.66	0.573	Eosinophil
LINC02972	Up	1.05	3.02×10^{-14}	8.40×10^{-16}	8.05	2	Yes	1.03	1.46×10^{-13}	1.10	0.568	lncRNA
SPNS3	Up	1.02	2.65×10^{-18}	1.62×10^{-20}	9.29	2	Yes	1.11	1.32×10^{-17}	0.75	0.568	Eosinophil
IL5RA	Up	1.02	6.12×10^{-21}	1.12×10^{-23}	10.03	2	Yes	1.32	3.54×10^{-22}	0.19	0.789	Eosinophil
MGAM2	Down	−1.00	1.54×10^{-11}	1.01×10^{-12}	−7.13	2	Yes	−0.98	5.78×10^{-11}	−1.09	0.568	Other
IGLV7-46	Down	−1.01	8.23×10^{-12}	5.03×10^{-13}	−7.22	2	Yes	−0.90	1.71×10^{-10}	−1.30	0.568	B cell
LINC02967	Down	−1.01	1.39×10^{-13}	4.98×10^{-15}	−7.83	2	Yes	−1.12	1.08×10^{-13}	−0.70	0.635	lncRNA
AIM2	Down	−1.01	1.06×10^{-21}	1.13×10^{-24}	−10.25	2	Yes	−1.04	3.93×10^{-21}	−0.93	0.568	B cell
SOCS3	Down	−1.02	2.50×10^{-21}	3.86×10^{-24}	−10.14	2	Yes	−1.22	4.47×10^{-22}	−0.45	0.667	Neutrophil
MCEMP1	Down	−1.02	7.81×10^{-19}	3.90×10^{-21}	−9.44	2	Yes	−1.17	1.28×10^{-19}	−0.59	0.684	Monocyte
ANXA3	Down	−1.03	5.76×10^{-16}	8.04×10^{-18}	−8.60	2	Yes	−1.09	1.12×10^{-15}	−0.87	0.570	Neutrophil
TLR5	Down	−1.03	1.68×10^{-25}	1.54×10^{-29}	−11.29	2	Yes	−1.05	3.85×10^{-25}	−0.99	0.568	Monocyte
C2	Down	−1.03	9.16×10^{-15}	2.06×10^{-16}	−8.22	2	Yes	−1.14	6.57×10^{-15}	−0.75	0.622	Monocyte
SHOC1	Down	−1.03	2.44×10^{-17}	2.24×10^{-19}	−9.00	2	Yes	−0.92	4.35×10^{-16}	−1.35	0.568	Neutrophil
CASP5	Down	−1.04	8.59×10^{-19}	4.41×10^{-21}	−9.42	2	Yes	−1.16	4.84×10^{-19}	−0.68	0.600	Monocyte
LINC03078	Down	−1.05	1.67×10^{-14}	4.20×10^{-16}	−8.13	2	Yes	−1.01	2.31×10^{-14}	−1.17	0.585	lncRNA
FGF13	Down	−1.05	4.87×10^{-19}	2.11×10^{-21}	−9.50	2	Yes	−1.22	1.92×10^{-19}	−0.59	0.614	Neutrophil

Gene symbol	Direction	Meta log_2FC	Meta FDR	Meta p	Stouffer Z	Studies	Concordant	GSE89403 log_2FC	GSE89403 FDR	Study cohort log_2FC	Study cohort FDR	HPA cell type
NRN1	Down	−1.05	1.46×10^{-18}	8.05×10^{-21}	−9.36	2	Yes	−1.24	2.88×10^{-19}	−0.52	0.672	T cell
FCGR1BP	Down	−1.05	2.28×10^{-18}	1.34×10^{-20}	−9.31	2	Yes	−1.05	2.69×10^{-18}	−1.08	0.571	Neutrophil
ITGA7	Down	−1.07	2.51×10^{-20}	6.88×10^{-23}	−9.85	2	Yes	−1.21	9.98×10^{-21}	−0.65	0.614	Other
CACNA1E	Down	−1.07	2.81×10^{-22}	1.86×10^{-25}	−10.43	2	Yes	−1.11	2.72×10^{-22}	−0.96	0.571	Other
GALNT14	Down	−1.08	4.57×10^{-19}	1.91×10^{-21}	−9.51	2	Yes	−1.05	5.15×10^{-18}	−1.15	0.568	Neutrophil
SLC26A8	Down	−1.09	2.13×10^{-21}	2.85×10^{-24}	−10.16	2	Yes	−1.10	1.14×10^{-20}	−1.05	0.568	Neutrophil
CAPN13	Down	−1.10	1.67×10^{-13}	6.13×10^{-15}	−7.80	2	Yes	−1.07	1.19×10^{-12}	−1.17	0.568	Other
MYO10	Down	−1.10	2.81×10^{-20}	8.05×10^{-23}	−9.83	2	Yes	−1.04	5.28×10^{-19}	−1.26	0.568	Monocyte
H2BP1	Down	−1.12	4.39×10^{-19}	1.78×10^{-21}	−9.52	2	Yes	−1.11	1.48×10^{-18}	−1.14	0.568	Other
SMTNL1	Down	−1.12	2.05×10^{-16}	2.54×10^{-18}	−8.73	2	Yes	−1.20	2.97×10^{-16}	−0.91	0.573	Neutrophil
GBP6	Down	−1.13	1.02×10^{-16}	1.12×10^{-18}	−8.82	2	Yes	−1.33	4.81×10^{-17}	−0.56	0.627	Neutrophil
CNGB1	Down	−1.15	3.85×10^{-18}	2.65×10^{-20}	−9.23	2	Yes	−1.33	7.97×10^{-19}	−0.63	0.675	Neutrophil
LINC01093	Down	−1.16	8.22×10^{-14}	2.73×10^{-15}	−7.90	2	Yes	−1.39	6.80×10^{-14}	−0.53	0.631	lncRNA
ZDHHC19	Down	−1.17	3.49×10^{-18}	2.20×10^{-20}	−9.25	2	Yes	−1.33	3.86×10^{-18}	−0.72	0.571	B cell
BATF2	Down	−1.19	3.62×10^{-14}	1.04×10^{-15}	−8.02	2	Yes	−1.33	2.81×10^{-14}	−0.80	0.628	Neutrophil
MAOB	Down	−1.20	2.91×10^{-13}	1.16×10^{-14}	−7.72	2	Yes	−1.25	1.20×10^{-12}	−1.07	0.568	Other
FCGR1A	Down	−1.21	3.89×10^{-20}	1.26×10^{-22}	−9.79	2	Yes	−1.29	3.38×10^{-20}	−0.99	0.575	Monocyte
PDCD1LG2	Down	−1.22	4.32×10^{-19}	1.71×10^{-21}	−9.52	2	Yes	−1.47	1.27×10^{-19}	−0.53	0.632	Monocyte
CARD17P	Down	−1.22	8.09×10^{-17}	8.79×10^{-19}	−8.85	2	Yes	−1.32	6.32×10^{-17}	−0.96	0.596	Other
KREMEN1	Down	−1.22	2.13×10^{-21}	2.97×10^{-24}	−10.16	2	Yes	−1.33	2.18×10^{-21}	−0.94	0.570	Neutrophil
C1QA	Down	−1.23	9.69×10^{-21}	1.85×10^{-23}	−9.98	2	Yes	−1.42	3.91×10^{-21}	−0.69	0.604	Monocyte
BASP1-AS1	Down	−1.23	1.06×10^{-21}	1.16×10^{-24}	−10.25	2	Yes	−1.22	1.30×10^{-20}	−1.26	0.568	lncRNA
CD274	Down	−1.23	1.09×10^{-19}	3.84×10^{-22}	−9.68	2	Yes	−1.29	3.81×10^{-19}	−1.07	0.568	Neutrophil
GBP1P1	Down	−1.24	1.45×10^{-12}	7.12×10^{-14}	−7.49	2	Yes	−1.37	3.35×10^{-12}	−0.88	0.573	Other
ANKRD22	Down	−1.25	9.52×10^{-15}	2.15×10^{-16}	−8.21	2	Yes	−1.39	6.47×10^{-15}	−0.86	0.628	Neutrophil
SERPING1	Down	−1.28	1.22×10^{-14}	2.95×10^{-16}	−8.18	2	Yes	−1.41	1.21×10^{-14}	−0.92	0.603	Neutrophil
LINC02751	Down	−1.31	1.92×10^{-17}	1.71×10^{-19}	−9.03	2	Yes	−1.19	6.14×10^{-16}	−1.64	0.568	lncRNA
LIPM	Down	−1.32	6.27×10^{-13}	2.75×10^{-14}	−7.61	2	Yes	−1.46	4.43×10^{-13}	−0.91	0.652	Other
SEPTIN4	Down	−1.41	1.25×10^{-16}	1.43×10^{-18}	−8.80	2	Yes	−1.68	5.86×10^{-17}	−0.64	0.631	T cell
C1QB	Down	−1.47	2.50×10^{-21}	3.78×10^{-24}	−10.14	2	Yes	−1.64	2.18×10^{-21}	−0.99	0.573	Monocyte
FAM20A	Down	−1.59	1.26×10^{-24}	3.71×10^{-28}	−11.00	2	Yes	−1.95	2.02×10^{-24}	−0.58	0.568	Neutrophil
C1QC	Down	−1.63	1.29×10^{-21}	1.52×10^{-24}	−10.23	2	Yes	−1.87	3.89×10^{-22}	−0.97	0.623	Monocyte
TMT1B	Down	−1.84	1.04×10^{-24}	2.30×10^{-28}	−11.05	2	Yes	−2.14	3.85×10^{-25}	−1.03	0.587	Other
CD177	Down	−2.41	5.44×10^{-21}	9.18×10^{-24}	−10.05	2	Yes	−2.51	3.24×10^{-20}	−2.11	0.568	Neutrophil

bioRxiv preprint doi: https://doi.org/10.64898/2026.10.25.761687; this version posted October 27, 2026. The copyright holder for this preprint (which was not certified by peer review) is the author/funder, who has granted bioRxiv a license to display the preprint in perpetuity. It is made available under a CC-BY 4.0 International license.

Supplementary Table S2. Differentially Expressed miRNAs with associated statistics.

All 7 DE-miRNAs with base mean, log2FC (apeglm-shrunk), log2FC standard error, p-value, Benjamini-Hochberg adjusted p-value, and significance classification. Differential expression performed using DESeq2 with paired design (~subject + condition) on study cohort small RNA-seq data (n = 11 pairs).

miRNA	Base mean	log_2FC	SE	p	FDR	Direction
miR-151a-5p/151b	9635.4	−0.64	0.14	1.60×10^{-7}	8.33×10^{-5}	Down
miR-139-5p	849.7	−0.75	0.23	5.20×10^{-5}	0.014	Down
miR-144-5p	142.4	0.94	0.36	2.13×10^{-4}	0.031	Up
miR-28-3p	719.6	−0.81	0.32	3.24×10^{-4}	0.034	Down
miR-144-3p	452.2	1.09	0.56	4.27×10^{-4}	0.037	Up
miR-32-5p	56.2	1.07	0.52	5.09×10^{-4}	0.038	Up
miR-4745-3p	73.3	−0.80	0.35	6.51×10^{-4}	0.042	Down

Supplementary Table S3. miRNA-mRNA target pairs identified by multiMiR.

Each row represents a miRNA-mRNA pair supported by either validated experimental evidence or computational prediction in the multiMiR database. miRNA log_2FC and mRNA log_2FC denote log2 fold change (week 4 vs baseline) for the miRNA (DESeq2, study cohort) and mRNA (limma, study cohort), respectively. mRNA log_2FC (meta-analysis) is the meta-analysis log_2FC. Inverse: Yes indicates opposing directionality of miRNA and mRNA fold change, consistent with canonical miRNA-mediated repression.

miRNA	Target	Evidence	multiMiR database(s)	miRNA log_2FC	mRNA log_2FC (study cohort)	mRNA log_2FC (meta-analysis)	Inverse
miR-139-5p	*C1QB*	Validated	tarbase	−0.746	−0.989	−1.468	No
miR-139-5p	*C2*	Validated	tarbase	−0.746	−0.753	−1.034	No
miR-139-5p	*FGF13*	Validated	tarbase	−0.746	−0.586	−1.052	No
miR-139-5p	*ITGA7*	Validated	tarbase	−0.746	−0.654	−1.067	No
miR-139-5p	*KREMEN1*	Validated	tarbase	−0.746	−0.936	−1.223	No
miR-139-5p	*MAOB*	Validated	tarbase	−0.746	−1.074	−1.201	No
miR-139-5p	*MYO10*	Validated	tarbase	−0.746	−1.261	−1.102	No
miR-139-5p	*PLAAT5*	Validated	tarbase	−0.746	0.400	1.155	Yes
miR-139-5p	*PMP22*	Validated	elmmo; miranda; mirdb; mirtarbase; tarbase; targetscan	−0.746	0.657	1.049	Yes
miR-139-5p	*SEPTIN4*	Validated	tarbase	−0.746	−0.636	−1.406	No
miR-139-5p	*SERPING1*	Validated	tarbase	−0.746	−0.919	−1.279	No
miR-144-5p	*HRK*	Validated	tarbase	0.941	0.900	1.286	No
miR-28-3p	*MYO10*	Validated	pita; tarbase	−0.813	−1.261	−1.102	No
miR-32-5p	*CD274*	Validated	tarbase	1.075	−1.075	−1.234	Yes
miR-32-5p	*CXCL8*	Validated	tarbase	1.075	1.580	1.061	No
miR-32-5p	*FGF13*	Validated	tarbase	1.075	−0.586	−1.052	Yes
miR-32-5p	*MYO10*	Validated	pita; tarbase	1.075	−1.261	−1.102	Yes
miR-32-5p	*PMP22*	Validated	tarbase	1.075	0.657	1.049	No
miR-144-3p	*ANXA3*	Predicted	microcosm	1.093	−0.867	−1.031	Yes
miR-144-3p	*MAOB*	Predicted	diana_microt; miranda	1.093	−1.074	−1.201	Yes
miR-144-3p	*MGAM2*	Predicted	diana_microt	1.093	−1.086	−1.004	Yes
miR-144-5p	*FGF13*	Predicted	miranda	0.941	−0.586	−1.052	Yes
miR-28-3p	*C2*	Predicted	microcosm	−0.813	−0.753	−1.034	No
miR-28-3p	*CD274*	Predicted	diana_microt	−0.813	−1.075	−1.234	No
miR-4745-3p	*ITGA7*	Predicted	diana_microt	−0.802	−0.654	−1.067	No

Supplementary Table S4. Differentially expressed tRFs with associated statistics and annotations.

tRF cluster name, base mean, log2FC (apeglm-shrunk), log2FC standard error, p-value, Benjamini-Hochberg adjusted p-value, biogenesis type (tRF-3, i-tRF, or 3' half) and dominant read sequence. All eight DE-tRFs were nuclear-encoded. Differential expression performed using DESeq2 with paired design on study cohort small RNA-seq data (n = 11 pairs).

tRF cluster	Biogenesis type	Base mean	log_2FC	SE	p	FDR	Dominant read sequence
Gly Comb 2	i-tRF	338.9	1.017	0.303	2.00×10^{-5}	0.002	AATTCTCGCCTGCCAC
Ala Comb 2	tRF-3	1267.0	0.970	0.352	1.31×10^{-4}	0.008	GCCCGCATCCTCCACCA
Ala Val Comb 2	tRF-3	6713.6	0.776	0.360	9.84×10^{-4}	0.022	TCCCCGGCACCTCCACCA
Gly Comb 1	tRF-3	4452.6	0.712	0.307	9.64×10^{-4}	0.022	CGATTCCCGGCCAATGCACCA
Leu Comb 8	tRF-3	1841.3	0.729	0.302	7.20×10^{-4}	0.022	ATCCCACTCCTGACACCA
Phe Comb 12	tRF-3	505.9	0.850	0.465	0.001	0.022	CCCGGGTTTCGGCACCA
Asp Comb 3	3' Half	1785.3	−0.654	0.318	0.002	0.034	CACGCGGGAGACCGGGGTTCGATTCCCCGACGGGGAGCCA
Cys Comb 5	tRF-3	108.1	0.711	0.490	0.003	0.037	TCCGGGTGCCCCCTCCA

bioRxiv preprint doi: https://doi.org/10.64898/2026.10.25.761687; this version posted October 27, 2026. The copyright holder for this preprint (which was not certified by peer review) is the author/funder, who has granted bioRxiv a license to display the preprint in perpetuity. It is made available under a CC-BY 4.0 International license.

Supplementary Table S5. Discrimination of week-4 sputum culture conversion by EARLY5 and published TB signatures.

Baseline samples from GSE89403 (n = 76; 18 fast converters). EARLY5 scores are out-of-fold LOOCV predictions (LOOCV accuracy 0.724; sensitivity 0.44, specificity 0.81, using a majority of gene-pair votes). Sweeney3 and RISK6 were scored with ssGSEA using TBSignatureProfiler; higher scores in both were associated with slower conversion. AUC 95% CIs were calculated with the DeLong method. Paired DeLong tests compared each comparator with EARLY5 after aligning the direction of the scores.

(a) Discrimination

Signature	n	AUC (95% CI)	Paired DeLong p vs EARLY5
EARLY5 (LOOCV)	76	0.761 (0.649–0.874)	—
Sweeney3	76	0.667 (0.520–0.814)	0.171
RISK6	76	0.628 (0.478–0.778)	0.113

(b) Out-of-fold EARLY5 classification of week-4 conversion (fast = positive class). TP, FN, FP, TN: true positives, false negatives, false positives, true negatives; PPV, NPV: positive and negative predictive values.

n	True fast	True slow	TP	FN	FP	TN	Sensitivity	Specificity	PPV	NPV	Accuracy
76	18	58	8	10	11	47	0.44	0.81	0.42	0.82	0.72

Supplementary Table S6. Cox proportional hazards models for time to sputum culture conversion.

The event is sputum culture conversion, assessed at weeks 4, 8, 12 and 24; all participants converted by week 24. HR > 1 indicates faster conversion. Predicted fast/slow is the out-of-fold EARLY5 classification (fast n = 19, slow n = 57). Ties were handled with the Efron method. Proportional hazards assumptions were checked with Schoenfeld residuals (global p = 0.93, 0.32 and 0.21 for the unadjusted, adjusted and continuous-score models). With exact handling of ties, HRs were 2.71 (1.16–6.36; p = 0.022) unadjusted, 2.58 (0.90–7.39; p = 0.077) after adjustment for MGIT TTP, and 1.11 (0.83–1.49; p = 0.469) for the continuous score.

Model	Term	n	HR (95% CI)	p	Ties
Unadjusted	Predicted fast vs slow	76	1.85 (1.08–3.16)	0.024	Efron
Unadjusted (MGIT complete cases)	Predicted fast vs slow	58	1.96 (1.06–3.65)	0.033	Efron
Adjusted for MGIT TTP	Predicted fast vs slow	58	1.72 (0.90–3.31)	0.102	Efron
Adjusted for MGIT TTP	MGIT TTP (per IQR)	58	1.30 (1.06–1.60)	0.013	Efron
Continuous score + MGIT TTP	EARLY5 score (per 0.2)	58	1.04 (0.85–1.28)	0.699	Efron
Continuous score + MGIT TTP	MGIT TTP (per IQR)	58	1.34 (1.05–1.70)	0.017	Efron
Unadjusted	Predicted fast vs slow	76	2.71 (1.16–6.36)	0.022	Exact
Adjusted for MGIT TTP	Predicted fast vs slow	58	2.58 (0.90–7.39)	0.077	Exact
Continuous score + MGIT TTP	EARLY5 score (per 0.2)	58	1.11 (0.83–1.49)	0.469	Exact

bioRxiv preprint doi: https://doi.org/10.64898/2026.10.25.761687; this version posted October 27, 2026. The copyright holder for this preprint (which was not certified by peer review) is the author/funder, who has granted bioRxiv a license to display the preprint in perpetuity. It is made available under a CC-BY 4.0 International license.

Supplementary Table S7. Stability of gene-pair selection across LOOCV folds.

In each of 76 leave-one-out folds, k-TSP selected up to five gene pairs from the training data (k = 5 in 65 folds, k = 4 in 9, k = 3 in 2). The five pairs that make up EARLY5 were the most frequently selected.

Gene pair	Folds selected (of 76)	%
DIS3L > DOCK4	76	100
COL9A2 > FAM241A	72	95
PIEZO1 > H2BC12	69	91
PDE4DIP > GRB10	67	88
PID1 > LYPLA1	54	71
PID1 > FCGR1BP	5	7
CYTH3 > GRB10	4	5
POU2F2 > CXCL16	3	4
RGS14 > SIRPB1	3	4
XPC > RNF11	3	4
COL9A2 > GCLM	2	3
ARHGEF2 > SIRPB1	1	1
GGA2 > H2BC12	1	1
KRT23 > LYPLA1	1	1
PDE4DIP > DCUN1D3	1	1
PID1 > ACSL3	1	1
PID1 > NINJ2	1	1
POU2F2 > SNX3	1	1
RTN1 > ACSL3	1	1
XPO5 > ACSL3	1	1

bioRxiv preprint doi: https://doi.org/10.64898/2026.10.25.761687; this version posted October 27, 2026. The copyright holder for this preprint (which was not certified by peer review) is the author/funder, who has granted bioRxiv a license to display the preprint in perpetuity. It is made available under a CC-BY 4.0 International license.

Supplementary Table S8. Immune cell type abundance at baseline and week 4 estimated by CIBERSORTx and MCP-counter.

Mean estimated abundance at baseline and week 4 in GSE89403 (n = 86 pairs). CIBERSORTx values are LM22 cell fractions (samples with CIBERSORTx-p < 0.05); MCP-counter values are scores estimated from TPM. Changes were assessed with paired Wilcoxon signed-rank tests, with Benjamini-Hochberg FDR correction within each method. — indicates cell types estimated at zero in all samples, which were not tested.

Method	Cell type	Mean baseline	Mean week 4	Paired Wilcoxon p	FDR
CIBERSORTx	B cells naive	0.0274	0.0205	0.001	0.005
CIBERSORTx	B cells memory	0.0314	0.0375	0.004	0.009
CIBERSORTx	Plasma cells	0.0039	0.0023	6.83×10^{-5}	3.41×10^{-4}
CIBERSORTx	T cells CD8	0.0907	0.1381	4.45×10^{-11}	8.91×10^{-10}
CIBERSORTx	T cells CD4 naive	0.0213	0.0222	0.911	0.959
CIBERSORTx	T cells CD4 memory resting	0.0014	0.0015	0.514	0.683
CIBERSORTx	T cells CD4 memory activated	0.0063	0.0025	0.001	0.005
CIBERSORTx	T cells follicular helper	0.0015	0.0019	0.556	0.683
CIBERSORTx	T cells regulatory (Tregs)	0.0829	0.1005	1.04×10^{-6}	6.97×10^{-6}
CIBERSORTx	T cells gamma delta	0.0000	0.0000	—	—
CIBERSORTx	NK cells resting	0.0652	0.0786	0.004	0.009
CIBERSORTx	NK cells activated	0.0152	0.0180	0.079	0.132
CIBERSORTx	Monocytes	0.0335	0.0320	0.629	0.698
CIBERSORTx	Macrophages M0	0.0001	0.0000	1.000	1.000
CIBERSORTx	Macrophages M1	0.0009	0.0005	0.015	0.030
CIBERSORTx	Macrophages M2	0.0193	0.0229	0.078	0.132
CIBERSORTx	Dendritic cells resting	0.0023	0.0021	0.536	0.683
CIBERSORTx	Dendritic cells activated	0.0039	0.0030	0.301	0.464
CIBERSORTx	Mast cells resting	0.0311	0.0297	0.581	0.683
CIBERSORTx	Mast cells activated	0.0000	0.0000	—	—
CIBERSORTx	Eosinophils	0.0000	0.0019	0.004	0.009
CIBERSORTx	Neutrophils	0.5616	0.4843	1.10×10^{-8}	1.10×10^{-7}
MCP-counter	B lineage	509.5	365.8	2.35×10^{-5}	4.71×10^{-5}
MCP-counter	CD8 T cells	48.3	66.9	4.39×10^{-12}	1.76×10^{-11}
MCP-counter	Cytotoxic lymphocytes	161.9	217.3	5.37×10^{-8}	1.43×10^{-7}
MCP-counter	Monocytic lineage	21.1	20.3	0.195	0.195
MCP-counter	Myeloid dendritic cells	4.5	4.7	0.160	0.183
MCP-counter	Neutrophils	536.8	493.0	0.003	0.003
MCP-counter	NK cells	4.2	4.9	0.002	0.003
MCP-counter	T cells	33.7	47.3	8.25×10^{-15}	6.60×10^{-14}
